# Novel evidence for oncogenic piRNA‐823 as a promising prognostic biomarker and a potential therapeutic target in colorectal cancer

**DOI:** 10.1111/jcmm.15537

**Published:** 2020-06-28

**Authors:** Junlan Feng, Muqing Yang, Qing Wei, Feifei Song, Youhua Zhang, Xiaodong Wang, Bin Liu, Jiyu Li

**Affiliations:** ^1^ Department of General Surgery School of Medicine Shanghai Tenth People's Hospital Tongji University Shanghai China; ^2^ Department of Pathology School of Medicine Shanghai Tenth People's Hospital Tongji University Shanghai China

**Keywords:** colorectal cancer, G6PD, glucose consumption, HIF‐1α, piRNA‐823

## Abstract

piRNA‐823 as a member of the piRNA family is reported to promote tumour cell proliferation in multiple myeloma and hepatocellular cancer. However, few studies on the function of piRNA‐823 in colorectal cancer (CRC). Our present study data showed that piRNA‐823 plays an oncogene role in CRC cells. Inhibition of piRNA‐823 can significantly inhibit the proliferation, invasion and apoptosis resistance of CRC cells. Mechanism studies have shown that piRNA‐823 inhibits the ubiquitination of hypoxia‐inducible factor‐1 alpha (HIF‐1α) by up‐regulating the expression of Glucose‐6‐phosphate dehydrogenase (G6PD) and ultimately up‐regulates the glucose consumption of carcinoma cells and inhibits the content of intracellular reactive oxygen species (ROS). Therefore, we speculate piRNA‐823 promotes the proliferation, invasion and apoptosis resistance of CRC cells by regulating G6PD/HIF‐1α pathway. In this study, we set up the cancer‐promoting function recovery experiment of piRNA‐823 by silencing G6PD gene to confirm the dominance of the above‐mentioned pathways. Using clinical samples, we found that overexpression of piRNA‐823 correlated with poor overall survival and predicted a poor response to adjuvant chemotherapy of patients with CRC. In a word, our research has further enriched the theory of piRNA‐823 promoting the progression of CRC, and laid a solid foundation for the development of piRNA‐823‐based gene therapy for CRC and its use as a promising prognostic biomarker in CRC patients.

## 1 INTRODUCTION

Colorectal cancer (CRC) is one of the most common malignant tumours in the world, and it is on the rise year by year. Finding new tumour markers and improving early clinical diagnosis are important means to improve the cure rate of patients with CRC. Piwi‐interacting RNA (piRNA) is a single‐stranded non‐coding RNA consisting of 23‐35 nucleotides, which was first discovered in mammalian germ cells. Because of its extensive cellular regulatory functions, it has become a star molecule in non‐coding RNA research. piRNA mainly exists in mammalian germ cells and stem cells. It binds with proteins belonging to Piwi subfamily to form piRNA complexes to regulate gene silencing pathways, maintain reproductive and stem cell functions and regulate the stability of translation of mRNA.^1^ In recent years, many studies have found that piRNA also exists in normal somatic cells (such as heart, brain, liver and other tissues) or cancer cells other than germ line cells, and widely participates in the occurrence and development of gastric cancer, liver cancer, lung cancer, lymphoma and other cancers, which is also closely correlated with central nervous system diseases, cardiac regeneration, etc.^2^, ^3^ Nevertheless, the clinical significance and biological mechanisms of piRNAs in the progression of CRC remain largely unknown.

In our present study, piRNA‐823, a family of piRNAs, was identified with high expression trends from normal colorectal tissues, adenoma and CRC by high‐throughput screening using the Arraystar Human piRNAs Array. Screening data show that the expression levels of piRNA‐823 are not only higher in colorectal cancer tissues than in adjacent tissues, but also positively correlated with tumour malignancy, suggesting that piRNA‐823 may be closely related to the progression of colorectal cancer. Therefore, the regulatory mode and action pathway of piRNA‐823 are worth studying. A group of screening data on gene expression profiles of HCT‐116 cells before and after piRNA‐823 intervention showed that molecule glucose‐6‐phosphate dehydrogenase (G6PD) gene related to glucose metabolism pathway in colorectal cancer cells changed significantly before and after piRNA‐823 intervention, and positively correlated with the content of piRNA‐823. Therefore, we speculated that piRNA‐823 may promote the occurrence and progression of colorectal cancer by enhancing the expression of downstream key molecule G6PD, which may be a key factor in the piRNA‐823 pathway to promote the progression of colorectal cancer.

## 2 MATERIALS AND METHODS

### 2.1 Clinical specimens collection

Five cases of CRC tissues and corresponding normal tissues used for piRNAs array analysis were obtained from the Department of GI Surgery, Shanghai Tenth People's Hospital, School of Medicine, Tongji University. 5 adenoma colorectal tissues were obtained from the Department of Digestive Endoscope. Validation using qRT‐PCR with CRC tissue samples was obtained from 176 patients from 2006 to 2012. The final date of follow‐up was 30 May 2017. A detailed clinical information for these patients is shown in Table S1. This study was approved by the hospital ethics committees, and written informed consents were obtained from patients for using their tissues and clinical information.

### 2.2 Microarray analysis

Total RNA from each sample was quantified by the NanoDrop ND‐1000, and RNA integrity was assessed by standard denaturing agarose gel electrophoresis. piRNA sample labelling was performed using a RNA ligase method as described in the method section. For microarray analysis, Agilent Array platform was employed. The labelled samples were hybridized onto Arraystar Human piRNA Array in Agilent's SureHyb Hybridization Chambers according to the manufacturer's standard protocols (Agilent Technologies). After having washed the slides, the arrays were scanned by the Agilent Scanner G2505C. Agilent Feature Extraction software (version 11.0.1.1) was used to analyse acquired array images. Quantile normalization and subsequent data processing were performed using the GeneSpring GX v11.5.1 software package (Agilent Technologies). After quantile normalization of the raw data, piRNAs that at least 5 out of 15 samples have flags in Present or Marginal (‘All Targets Value’) were chosen for further data analysis. Significant differentially expressed genes between two samples were identified through Fold Change filtering. Significant differentially expressed genes between two groups were identified through Volcano Plot filtering. Finally, hierarchical clustering was performed to show the distinguishable piRNAs expression pattern among samples.

### 2.3 Cell culture

HCT‐116, Lovo and 293T cells, purchased from the Cell Bank of the Chinese Academy of Sciences, were maintained in Dulbecco's Modified Eagle Medium (DMEM, Invitrogen) supplemented with 10% foetal bovine serum (FBS, Invitrogen) at 37°C under 5% CO_2_. The cells were passaged at the coverage of 70% using 0.25% Typsin (Invitrogen).

### 2.4 Lentivirus packaging

A siRNA sequence complementarily binding to G6PD (NM_000402, NM_001042351) was chosen. The target sequences of siRNA (5′‐GGTCAAGGTGTTGAAATGC‐3′) are homologous, and the oligonucleotide templates of these shRNAs were chemically synthesized and cloned into the linear pSIH1‐H1‐copGFP shRNA Vector (System Biosciences, CA, USA) which was obtained through digestion by BamHI and EcoRI (Takara, Dalian, China) and purification by agarose gel electrophoresis. An invalid siRNA sequence (5′‐GAAGCCAGATCCAGCTTCC‐3′) was used as a negative control (NC). Sequencing was used to confirm the vectors constructed (pSIH1‐shRNA‐G6PD and pSIH1‐NC). Chemically, synthesize complementary double‐stranded piRNA823 and piRNA823 sponge sequences with adding BamHI and EcoRI restriction sites at both ends. piRNA823‐forward, 5′‐GATCCAGCGTTGGTGGTATAGTGGTGAGCATAGCTGCG‐3′; piRNA‐823‐reverse, 5′‐AATTCGCAGCTATGCTCACCACTATACCACCAACGCTG‐3′;piRNA‐823 sponge‐forward, 5′‐GATCCAGCGTTGGTGGTATAGTGGTGAGCATAGCTGCTATACAGCGTTGGTGGTATAGTGGTGAGCATAGCTGCACATCAGCGTTGGTGGTATAGTGGTGAGCATAGCTGCG‐3′; piRNA‐823 sponge‐reverse, 5′‐AATTCGCAGCTATGCTCACCACTATACCACCAACGCTGATGTGCAGCTATGCTCACCACTATACCACCAACGCTGTATAGCAGCTATGCTCACCACTATACCACCAACGCTG‐3′; Two complementary DNA annealed to form a double strand and cloned into a pcDH1 lentiviral expression vector (System Biosciences). The recombinant vector was named pcDH1‐piRNA‐823 and pcDH1‐Sponge‐piRNA‐823. All the recombinant vectors were sequenced, and plasmid DNA was prepared using an EndoFree Plasmid Kit (12362, Qiagen).

One day before transfection, 293T cells were seeded into 10‐cm dishes (Corning). 2 μg of each pSIH1‐shRNA‐G6PD vector or pSIH1‐NC or pcDH1‐piRNA‐823 or pcDH1‐Sponge‐piRNA‐823 and 10 μg pPACK Packaging Plasmid Mix (System Biosciences) were co‐transfected using Lipofectamine 2000 (Invitrogen) in accordance with the manufacturer's protocol. The medium was replaced with DMEM plus 1% FBS. Forty eight hours later, the supernatant was harvested, and then cleared by centrifugation at 5000 *g* at 4°C for 5 minutes and passed through a 0.45 µm PVDF membrane (Millipore). The titre of virus was determined by gradient dilution. The packaged lentiviruses were named as Lv‐shRNA‐G6PD, Lv‐NC, Lv‐piRNA823 and Lv‐Sponge‐piRNA823.

### 2.5 Gene intervention via the lentiviral pathway

HCT‐116 and Lovo in the logarithmic phase were seeded in 6‐well plates at 2 × 10^5^ cells/well. One day later, lentivirus (Lv‐NC or Lv‐priRNA‐823 or Lv‐Sponge‐priRNA‐823 or Lv‐shRNA‐G6PD) were added at a multiplicity of infection (MOI) of 10. The infection efficiency was evaluated by observing the fluorescence of green fluorescent protein (GFP) 72 hours after infection. After determining the efficiency of the gene intervention, total RNA and protein were isolated from the cells and subjected to real‐time PCR for the measurement of the levels of priRNA‐823 and Western blotting for the measurement of the levels of G6PD, respectively.

### 2.6 Cell counting kit‐8 (CCK‐8) assay

HCT‐116 and Lovo were trypsinized and seeded into 96‐well plates at a density of 1 × 10^5^ cells per well 72 hours after being infected with the recombinant lentiviruses (Lv‐NC or Lv‐Sponge‐priRNA‐823). The cells were cultured under normal conditions, and cell viability was detected by using a cell counting kit‐8 assay (CCK‐8) at 24, 48, and 72 hours. Briefly, 10 µL of CCK‐8 solution (CK04, Dojindo, Japan) was added, and then the cells were cultured under normal conditions for an additional 4 hours. Then, the absorbance at 450 nm was measured.

### 2.7 Cell invasion assay

Cell invasion experiments were performed using the QCMTM 24‐well Fluorimetric Cell Invasion Assay kit (ECM554, Chemicon International) according to the manufacturer′s instructions. The kit used an insert polycarbonate membrane with an 8‐μm pore size. The insert was coated with a thin layer of EC Matrix™ that occluded the membrane pores and blocked the migration of non‐invasive cells. Culture medium (500 μL) supplemented with 10% FBS was used as a chemoattractant. Cells that migrated and invaded the underside of the membrane were fixed in 4% paraformaldehyde. The invading cells were stained with DAPI, and the number was then determined by fluorescence and reported as the relative fluorescence units (RFUs). The grouping was the same as in the proliferation assay. HCT‐116 and Lovo 72 hours after being infected with the recombinant lentiviruses were seeded to transwell at 2 × 10^5^ cells/well and 48 hours after seeding, cell invasion assay was performed.

### 2.8 Apoptosis assay

HCT‐116 and Lovo 72 hours after being infected with the recombinant lentiviruses were seeded to 6‐well plates at 2 × 10^5^ cells/well and 48 hours after seeding, cells were stained with Annexin V: FITC Apoptosis Detection KitII (BD). Cells were made into suspensions by trypsinization and washed with dPBS and suspended in 500 μL binding buffer and added with 5 μL Annexin V‐FITC in dark for 10 minutes. Cells were then stained with 5 μL Propidium Iodide for 5 minutes. Apoptosis was analysed on BD‐FACS Calibur using FITC (FL1) channel and PI (FL2) channel at an excitation wavelength at 488 nm.

### 2.9 Glucose consumption assay

Glucose consumption was determined as described with some modifications.^4^ HTC‐116 and Lovo cells infected with Lv‐Sponge‐piRNA‐823 or Lv‐NC or without infection were cultured in Dulbecco's modified Eagle's medium (DMEM) containing 10% (vol/vol) foetal bovine serum (FBS), and subsequently serum‐starved overnight. The glucose in the medium was determined by the glucose oxidase method. Glucose consumption % = (glucose concentrations of blank wells—glucose concentrations of assay wells)/ glucose concentrations of blank wells × 100.

### 2.10 Detection of intracellular ROS

The total intracellular ROS generation was measured using H2DCFDA, as reported by a published study.^5^ Briefly, HTC‐116 and Lovo cells infected with Lv‐Sponge‐piRNA‐823 or Lv‐NC or without infection were seeded (5 × 104 cells/well) into black 96‐well plates and maintained to attach at 37°C in 5% CO_2_ for 48 hours. After that, the cells were labelled with H2DCFDA solution at 5 μmol/L in DMF. After that, the fluorescence intensity was measured in a Synergy H1 Fluorescence Spectrophotometer (BioTek), at the excitation and emission wavelengths of 495 and 527 nm, respectively. After that, the ROS levels were measured using Cellular ROS/Superoxide Detection Assay Kit (Abcam).

### 2.11 Measuring the half‐life of G6PD

HTC‐116 cells in logarithmic phase were seeded to 6‐well plates and cultured under normal conditions overnight. Cells were divided into two groups, piRNA‐823 overexpression and suppression groups (infected with Lv‐piRNA‐823 or Lv‐Sponge‐piRNA‐823) and added with 50 μmol/L mg132 or 100 μg/mL CHX(sigma) and incubated under normal conditions for 0, 1, 2, 4 or 8 hours. Cells were collected and subjected to Western blotting for HIF‐1α.

### 2.12 Real‐Time‐PCR

To test the piRNA‐823 levels, total RNA (2 μg) was used for cDNA preparation with a M‐MLV reverse transcription kit and specific primers U6 snRNA (NM_001101. 3): 5′‐TACCTTGCGAAGTGCTTAAAC‐3′, and piRNA‐823:5′‐ GTCGTATCCAGTGCGTGTCGTGGAGTCGGCAATTGCACTGGATACGAGCAGC‐3′. RNA contents were detected using fluorescent dye PCR (Takara BIO) in accordance with the manufacturer's instructions. The following primers were used for quantification of human U6 snRNA and piRNA‐823: U6 snRNA: 5′‐GTGCTCGCTTCGGCAGCACAT‐3′ and 5′‐TACCTTGCGAAGTGCTTAAAC‐3′, which produced a segment of 112 bp; and piRNA‐823:5′‐GCCGGCGCCCGAGCTCTGGCTC‐3′ and 5′‐ GCGTTGGTGGTATAGTGGTGA‐3′, which produced a segment of 92 bp. The PCR systems were Takara SYBR Premix Ex Tap 10 μL, forward and reverse primers (20 µmol/L) 0.2 μL each and cDNA 2 μL added with dH_2_O to 20 μL. The cycling parameters were 40 cycles of denaturation at 95°C for 10 seconds, annealing at 60°C for 20 seconds and extension at 72°C for 20 seconds. U6 snRNA was used as a reference to normalize the hsa‐miRNA‐29a level using the 2^ΔΔCt^ method. Each RNA sample was run in triplicate.

### 2.13 Western blotting

The total protein was extracted from cells using the M‐PER mammalian protein extraction reagent (78501, Pierce, IL, USA). Equal amounts of total protein (15 μg) were loaded onto SDS‐PAGE gels (11%) and transferred onto nitrocellulose membranes. The blots were probed with the primary antibodies against human G6PD (1:400), STATS( 1:500), CyclinD1(1:500), Bcl‐2(1:400), HIF‐1α (1:500), and β‐actin (1:1200) (Abcam), followed by probing with the secondary HRP‐conjugated anti‐rabbit/mouse antibody (Abcam). After washing, the bands were detected by chemiluminescence and imaged with X‐ray films. β‐actin was used as an endogenous reference for normalization.

### 2.14 Statistical analysis

The data are shown as the mean ± SD of three independent experiments. All statistical data were analysed using SPSS GradPack version 20.0 statistical software (IBM Corp.) and GraphPad Prism 7.0 (GraphPad Software, Inc). Comparisons between groups were analysed using a two‐tailed Student's *t* test or one‐way ANOVA with a post hoc Tukey's test. Differences were considered to be statistically significant when **P < .05.

## 3 RESULTS

### 3.1 piRNA‐823 expression is significantly up‐regulated in the CRC progression process

**FIGURE 1:**
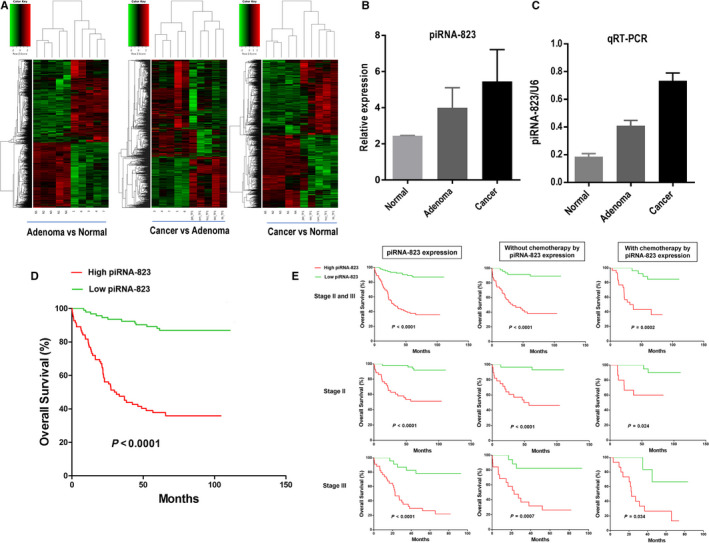
Clinical significance of piRNA‐823 expression in CRC patients. A‐C, piRNA array assays (A‐B) and qRT‐PCR validation (B) for piRNA‐823 expression in CRC, adenoma and matched normal colorectal tissues. C, High piRNA‐823 expression in tumours is associated with a reduced survival. D, Association of piRNA‐823 expression with the prognosis and chemotherapy outcome in the CRC patients with TNM stage II and III disease

To search for potential piRNAs in the course of colorectal malignant transformation, we globally analysed the piRNA expression profiles of normal colorectal tissues, colorectal adenoma tissues and CRC tissues using Arraystar Human piRNAs Array (Figure 1A). We focused on only one piRNA (piRNA‐823), a family of piRNAs, its expression level with high expression trends from normal colorectal tissues, adenoma and CRC (*P* = .007, One‐way ANOVA analysis; Figure 1B). To confirm the altered expression of piRNA‐823 in CRC, validation experiments were carried out by qRT‐PCR. The similar expression pattern was also confirmed (*P* < .001, One‐way ANOVA analysis; Figure 1C). Screening and validation data show that the expression levels of piRNA‐823 are not only higher in colorectal cancer tissues than in adjacent tissues, but also positively correlated with tumour malignancy, suggesting that piRNA‐823 may be closely related to the progression of colorectal cancer. Therefore, the regulatory mode and action pathway of piRNA‐823 are worth studying.

### 3.2 The association of the piRNA‐823 with the prognosis and therapeutic outcome of CRC

To investigate the association between piRNA‐823 expression and the clinicopathological characteristics of CRC patients, we examined a panel of 176 CRC tissues and paired adjacent non‐tumour tissues using qRT‐PCR. To correlate piRNA‐823 expression levels with clinicopathologic characteristics, the 176 CRC patients were classified into a relatively high group and a relatively low group using the median expression level of piRNA‐823 in CRC tissues as a cut‐off value. The current results showed that patients with high piRNA‐823 expression in CRC had significantly worse prognosis than those with low piRNA‐823 expression (Figure 1D, *P* < .001, Kaplan‐Meier test). In the univariate analysis, high expression of piRNA‐823 in tumours (HR, 7.49; 95% CI, 3.98‐14.08; *P* < .001), TNM staging (HR, 2.70; 95% CI, 1.64‐4.44; *P* < .001) and lymph node metastasis (HR, 2.32; 95% CI, 1.79‐4.17; *P* < .001) was significantly associated with survival, while age, gender, tumour location, differentiation and tumour size were not (Table 1). Multivariate analyses revealed that the level of piRNA‐823 expression is an independent prognostic factor for overall survival (HR, 8.02; 95% CI, 3.86‐16.68; *P* < .001) (Table 1). Taken together, these data demonstrated that an important role for piRNA‐823 in CRC carcinogenesis and progression and might be as a potential prognostic biomarker for CRC patients.

**TABLE 1:** Univariate and multivariate cox regression analyses of piRNA‐823 expression levels and overall cancer survival in colorectal cancer

Characteristic	Univariate analysis		Multivariate analysis	
	HR (95% CI)	*P* value	HR (95% CI)	*P* value*
piRNA‐823 expression				
Low	1.0 (Reference)	<.001	1.0 (Reference)	<.001
High	7.49 (3.98‐14.08)		8.02 (3.86‐16.68)	
TNM stage				
I‐II	1.0 (Reference)	<.001	1.0 (Reference)	<.001
III‐IV	2.70 (1.64‐4.44)		2.66 (1.52‐4.65)	
N stage				
N0	1.0 (Reference)	<.001	1.0 (Reference)	.145
N1‐N2	2.32 (1.79‐4.17)		1.49 (0.87‐2.53)	
Age				
<50	1.0 (Reference)	.414	1.0 (Reference)	.985
≥50	0.66 (0.24‐1.81)		0.99 (0.33‐2.99)	
Sex				
Women	1.0 (Reference)	.152	1.0 (Reference)	.561
Men	0.70 (0.42‐1.14)		1.17 (0.69‐1.99)	
Tumor location				
Colon	1.0 (Reference)	.692	1.0 (Reference)	.307
Rectum	1.11 (0.66‐1.89)		1.35 (0.76‐2.38)	
Tumor size				
<5 cm	1.0 (Reference)	.963	1.0 (Reference)	.598
≥5 cm	0.99 (0.59‐1.65)		1.16 (0.67‐2.01)	
Differentiation				
Good	1.0 (Reference)	.438	1.0 (Reference)	.952
Moderate or poor	2.19 (0.30‐15.76)		1.07 (0.13‐8.46)	

Abbreviations: CI, confidence interval; HR, hazard ratio.

* A *P* value of <.05 was considered significant.

To examine whether piRNA‐823 expression can predict response to adjuvant therapy, we analysed the associations between piRNA‐823 expression and the therapeutic outcomes in stage II and stage III CRC patients treated with adjuvant chemotherapy. The current results indicated that high piRNA‐823 expression was associated with a poor prognosis in stage II (*P* < .0001) and stage III (*P* < .0001) patients (Figure 1E). For individuals who received adjuvant therapy, high piRNA‐823 expression was associated with a poor therapeutic outcome in the patients with stage II and III cancer (*P* < .0001), and patients with stage II cancer alone (*P* < .0001) or patients with stage III cancer alone (*P* = .0007, Figure 1E). A multivariate Cox regression demonstrated that high piRNA‐823 expression predicted poor prognosis (HR, 12.76; 95% CI, 3.94‐41.32; *P* < .001) and treatment with adjuvant chemotherapy was associated with beneficial survival (HR, 0.42; 95% CI, 0.15‐0.69; *P* = .007) independent of other clinical covariates (Table 2). Therefore, piRNA‐823 expression levels served as an independent predictor of the response to adjuvant chemotherapy.

**TABLE 2:** Univariate and multivariate Cox proportional‐hazard regression analyses of piRNA‐823 expression, receipt of adjuvant chemotherapy and cancer survival in colorectal cancer with stage II or III

Characteristic	Univariate analysis		Multivariate analysis	
	HR (95% CI)	*P* value	HR (95% CI)	*P* value^a^
piRNA‐823 expression		<.001		<.001
Low	1.0 (Reference)		1.0 (Reference)	
High	7.97 (3.89‐16.31)		12.76 (3.94‐41.32)	
Adjuvant chemotherapy		.031		.007
Did not receive	1.0 (Reference)		1.0 (Reference)	
Received	0.43 (0.20‐0.92)		0.42 (0.15‐0.69)	
TNM stage		.001		.132
II	1.0 (Reference)		1.0 (Reference)	
III	2.49 (1.47‐4.23)		2.04 (0.81‐5.15)	
N stage		.002		.888
N0	1.0 (Reference)		1.0 (Reference)	
N1‐N2	3.01 (1.49‐6.09)		1.10 (0.30‐4.01)	
Age		.424		.412
<50	1.0 (Reference)		1.0 (Reference)	
≥50	0.62 (0.19‐1.99)		0.47 (0.079‐2.82)	
Sex		.138		.222
Women	1.0 (Reference)		1.0 (Reference)	
Men	0.67 (0.40‐1.14)		1.73 (0.72‐4.16)	
Tumor location		.503		.180
Colon	1.0 (Reference)		1.0 (Reference)	
Rectum	1.21 (0.69‐2.12)		1.90 (0.74‐4.86)	
Tumor size		.788		.246
<5 cm	1.0 (Reference)		1.0 (Reference)	
≥5 cm	0.93 (0.54‐1.59)		0.56 (0.21‐1.49)	
Differentiation		.596		.882
Good	1.0 (Reference)		1.0 (Reference)	
Moderate or Poor	1.71 (0.24‐12.35)		1.18 (0.12‐21.35)	

Note

*P* value of <.05 was considered significant.

Abbreviations: CI, confidence interval; HR, hazard ratio.

### 3.3 Knock‐down of piRNA‐823 inhibits the malignant characteristics of CRC cells

**FIGURE 2:**
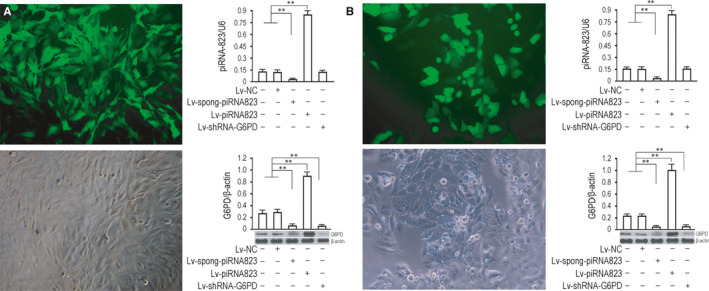
Lentivirus‐mediated gene intervention. A, HCT‐116 and B, Lovo. GFP expression 72 h after cells was infected with recombinant virus. The infection rate was estimated by dividing the number of the cells expressing GFP with the number of all the cells in each view. For statistics, five views were randomly selected, and the mean was calculated (left). Real‐Time PCR assay for piRNA823, U6 served as internal reference for the determination of, and the content of the uninfected group was used for normalization (left, upper). For Western blotting. β‐actin was used as the internal control protein, the target band sizes of the G6PD and β‐actin proteins were 53, and 43 kD, respectively. **P < .01, *P < .05. The tests were carried out on three biological triplicates, and data are expressed as the mean ± SD

To evaluate the oncogenic properties and effects of piRNA‐823 on CRC cells, we established CRC cell lines with effective piRNA‐823 gene intervention through lentivirus pathway. According to multiplicity of infection (MOI) = 10, lentivirus was added to colorectal cancer cell HCT‐116. After 72 hours of virus infection, we could roughly estimate that the viral infection efficiency of cells was close to 100% by comparing the number of cells expressing GFP with the total number of cells under microscope (Figure 2A, left). Real‐time PCR results showed that the relative content of piRNA‐823 in cells decreased significant 72 hours after Lv‐spong‐piRNA‐823 virus infection (*P* < .01, vs uninfected control group or NC control group). 72 hours after Lv‐piRNA‐823 infection, the relative content of piRNA‐823 in cells increased significantly (*P* < .01, vs‐uninfected control group). In Lv‐NC infected group or NC control group, the relative content of piRNA‐823 did not change significantly (*P* > .05, vs uninfected control group).

**FIGURE 3:**
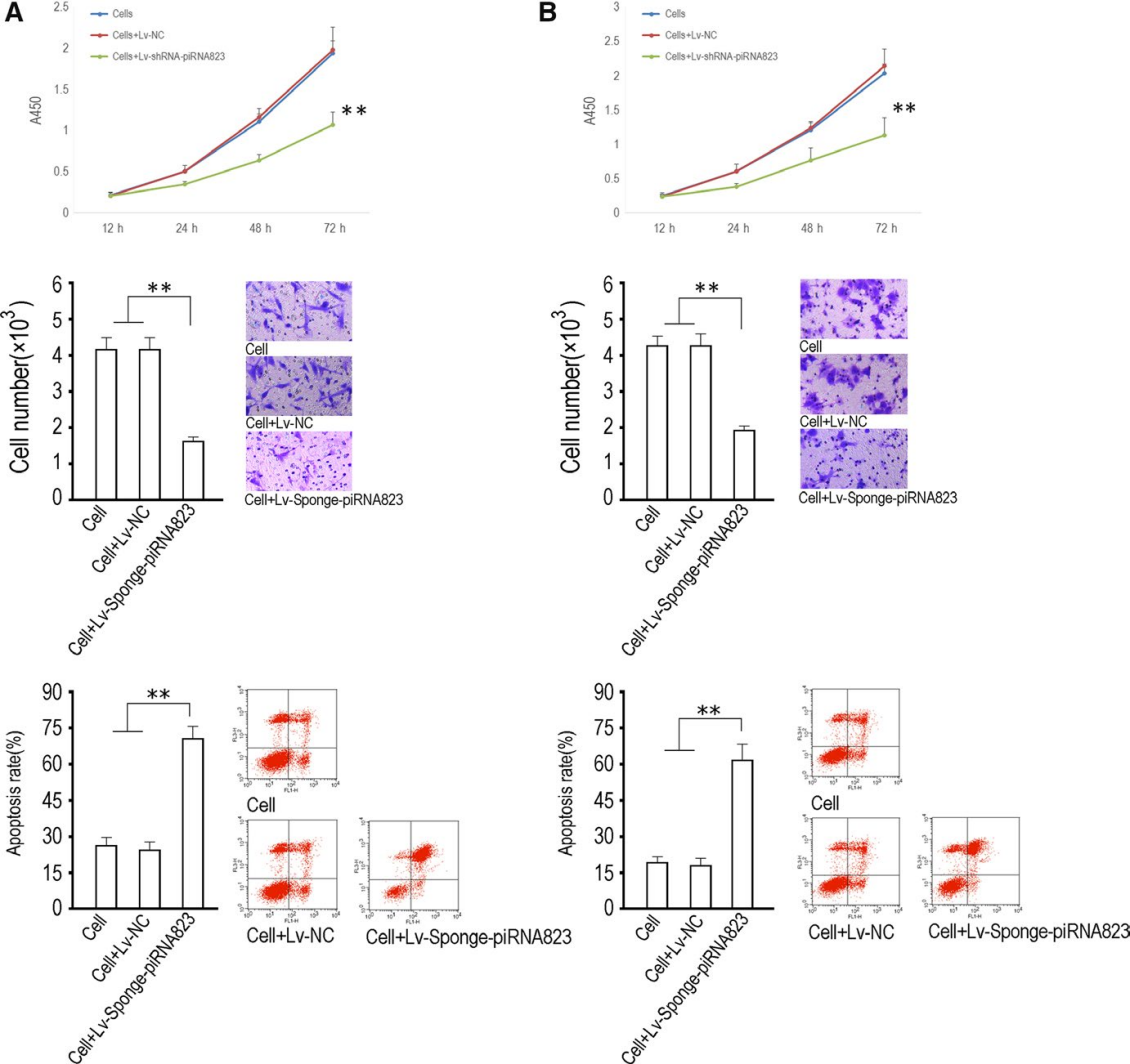
Inhibition of piRNA‐823 on the proliferation, invasion and apoptosis of HTC‐116 and Lovo cells. A, HTC‐116 and B, Lovo cells were infected with the indicated lentiviruses and then seeded into 96‐well plates and subjected to a cell vitality assay at the indicated times. Invasion data from the HTC‐116 and Lovo cells 72 h after being infected with the indicated viruses, as determined by a transwell method. The crystal violet‐stained cells are the cells that passed through the membrane, and the counts reflect the number of the stained cells that passed through the membrane, which were estimated using the absorbance and the standard curve. The x‐coordinate represents the cell grouping, and the y‐coordinate represents the cell number. C, Flow detection of apoptosis. The abscissa was FITC and the ordinate was PI fluorescence signal. The lower right quadrant and the upper right quadrant respectively indicated the proportion of apoptotic cells in the early and late stages. The statistical apoptosis rate between groups was the sum of the early and late apoptosis rates. All data were expressed with average value plus or minus variance. In this experiment, 3 biological repetitions were set (n = 3), **P < .01, *P < .05

In the functional experiment, proliferation activity assay data showed that piRNA‐823 inhibition significantly inhibited the logarithmic proliferative activity of HCT‐116 and Lovo cells (*P* < .01, vs cell control group or NC control group, 72 hours) (Figure 3A,B). Invasion assay showed that piRNA‐823 inhibition significantly inhibited the invasion of HCT‐116 and Lovo cells (*P* < .01, vs cell control group or NC control group) (Figure 3A,B). Apoptosis assay data showed that piRNA‐823 inhibition significantly up‐regulated apoptosis of HCT‐116 and Lovo cells (*P* < .01, vs cell control group or NC control group) (Figure 3A,B).

### 3.4 piRNA‐823 promotes CRC progression by up‐regulating G6PD expression

To understand the mechanism of piRNA‐823 regulation in CRC, a group of screening data on gene expression profiles of HCT‐116 cells before and after piRNA‐823 intervention showed that molecule glucose‐6‐phosphate dehydrogenase (G6PD) gene related to glucose metabolism pathway in colorectal cancer cells changed significantly before and after piRNA‐823 intervention and positively correlated with the content of piRNA‐823 (Table S2). Therefore, we speculated that piRNA‐823 may promote the occurrence and progression of colorectal cancer by enhancing the expression of downstream key molecule G6PD, which may be a key factor in the piRNA‐823 pathway to promote the progression of colorectal cancer.

In Lv‐shRNA‐G6PD infected group, the content of piRNA‐823 did not change significantly (*P* > .05, vs uninfected control group or NC control group) (Figure 2A, upper right). Western blotting data showed that the expression of G6PD protein in cells decreased significantly 72 hours after recombinant virus Lv‐spong‐piRNA‐823 infection (*P* < .01, vs‐uninfected control group or NC control group). The expression of G6PD protein in cells increased significantly 72 hours after Lv‐piRNA‐823 infection (*P* < .01, vs‐uninfected control group or NC control group). There was no significant change in G6PD protein expression in cells infected by Lv‐NC (*P* > .05, vs uninfected control group), but the expression of G6PD protein in cells infected by Lv‐shRNA‐G6PD decreased significantly (*P* < .01, vs uninfected control group or NC control group) (Figure 2A, lower right). The experimental data in Lovo cells were consistent with HCT‐116 (Figure 2B).

### 3.5 Overexpression and inhibition of piRNA‐823 can up‐regulate or inhibit ROS content and glucose consumption in colorectal cancer cells

**FIGURE 4:**
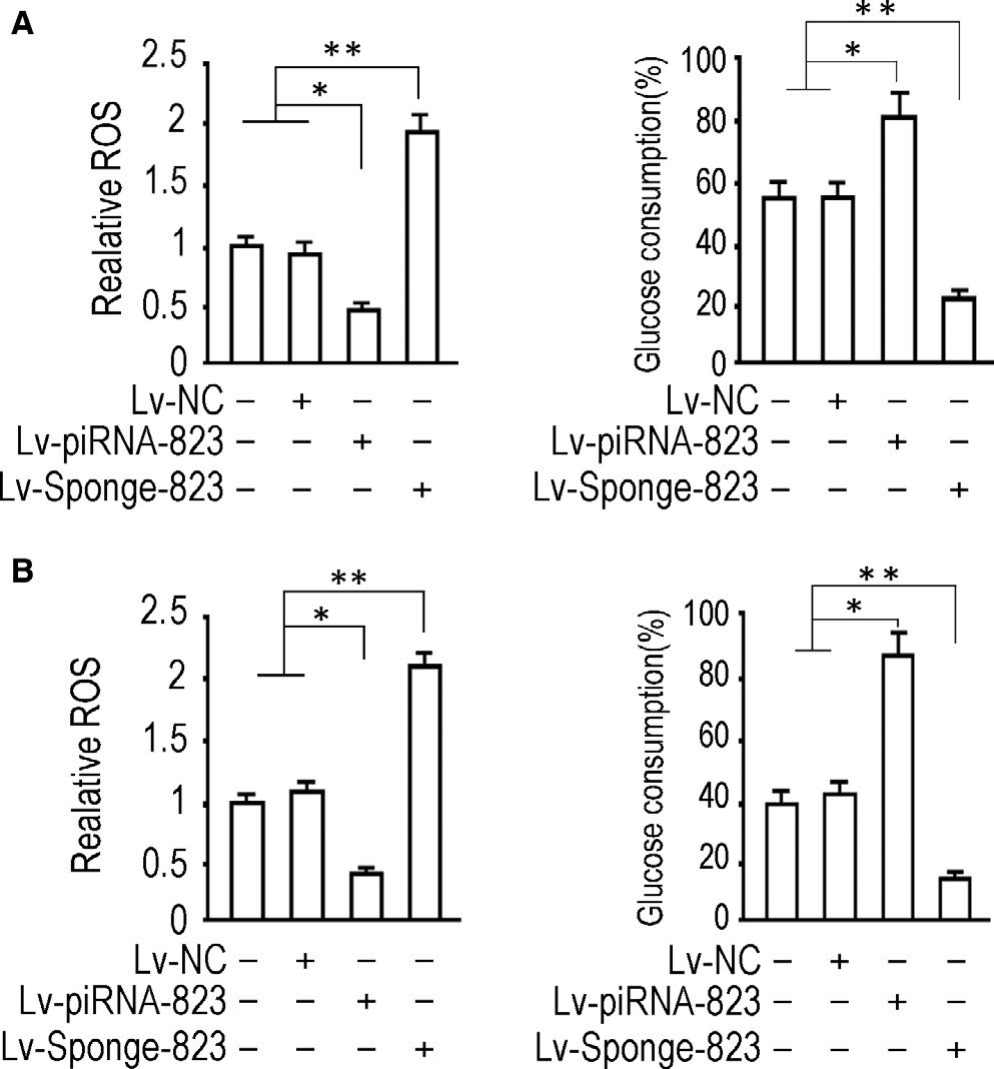
Effect of piRNA‐823 intervention on ROS activity and glucose consumption in HCT‐116 and Lovo cells. A, HCT‐116, B, Lovo. The ROS content was measured 72 h after the virus infection, and the cell test data of each group were homogenized according to the control group (left). The glucose in the medium was determined by the glucose oxidase method. Calculation of percentage glucose consumption (GC%) is described in Materials and Methods (right). **P < .01, *P < .05. The tests were carried out on three biological triplicates, and data are expressed as the mean ± SD

ROS activity test data showed that 72 hours after Lv‐piRNA‐823 infection, ROS content in HCT‐116 cells decreased significantly (*P* < .05, vs‐uninfected control group or NC control group); 72 hours after Lv‐spong‐piRNA‐823 infection, ROS content in cells of two groups increased significantly (*P* < .01, vs‐uninfected control group or NC control group); Lv‐NC infection had no significant effect on ROS content in cells (*P* > .05, vs uninfected control group) (Figure 4A, left). Glucose uptake experiments showed that 72 hours after Lv‐piRNA‐823 infection, the content of glucose in HCT‐116 cells increased significantly (*P* < .05, vs‐uninfected control group or NC control group), 72 hours after Lv‐spong‐piRNA‐823 infection, intracellular glucose exchange decreased significantly (*P* < .05, vs‐uninfected control group or NC control group). Lv‐NC infection had no significant effect on glucose uptake in tumour cells (*P* > .05, vs uninfected control group) (Figure 4A, Right). The experimental data in Lovo cells were consistent with HCT‐116 (Figure 4B).

### 3.6 piRNA‐823 up‐regulates the expression of functional proteins CyclinD1, STAT3 and Bcl‐2 by up‐regulating G6PD expression

**FIGURE 5:**
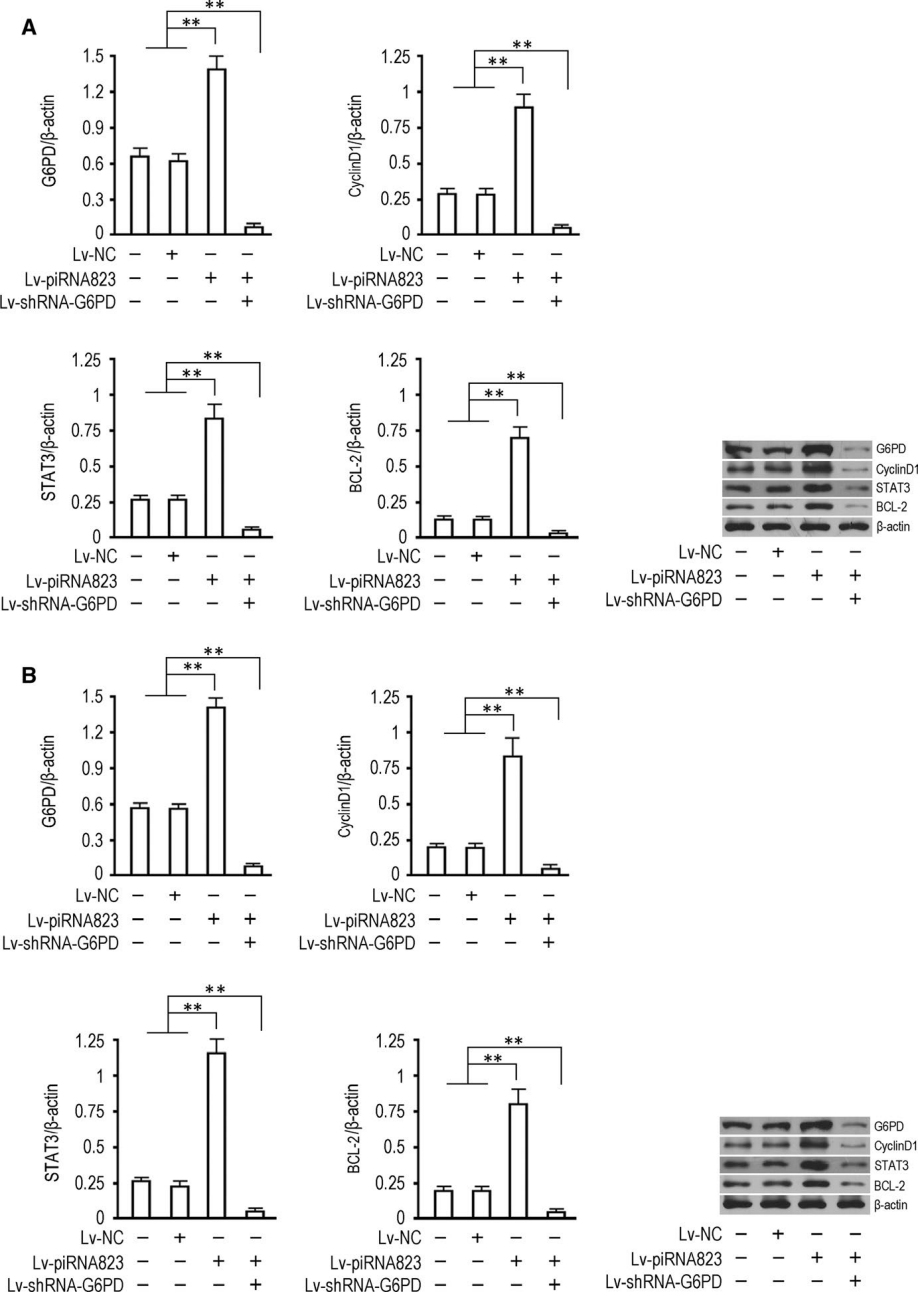
piRNA‐823 up‐regulates the expression of functional proteins CyclinD1, STAT3 and Bcl‐2 through G6PD. A, HCT‐116, B, Lovo. Western blotting was performed 72 h after virus infection. The protein was detected by β‐actin, and the size of G6PD, CyclinD1, STAT3, Bcl‐2 and β‐actin was 53 kD, 33 kD, 90 kD, 26 kD and 43 kD. All data were expressed as mean ± SD, and the experiment was set to 3 biological replicates (n = 3). **P < .01, *P < .05

Western blotting data showed that 72 hours after Lv‐piRNA82 infection, the expression of G6PD protein was significantly enhanced in two groups (*P* < .01, vs uninfected control group or NC control group). The expression of G6PD protein in Lv‐piRNA823 infected cells increased significantly 72 hours after infection (*P* < .01, vs uninfected control group or NC control group). Lv‐shRNA‐G6PD could significantly block the up‐regulation of G6PD protein expression in Lv‐piRNA823 infected cells (*P* < .01, vs Lv‐piRNA823 infected group). The expression trend of functional proteins CyclinD1, STAT3 and BCL‐2 related to cell proliferation, invasion and apoptosis in each group was completely consistent with that of G6PD (Figure 5A,B).

### 3.7 Effect of piRNA‐823 on the ubiquitylation of HIF‐1α in HCT‐116 cells

**FIGURE 6:**
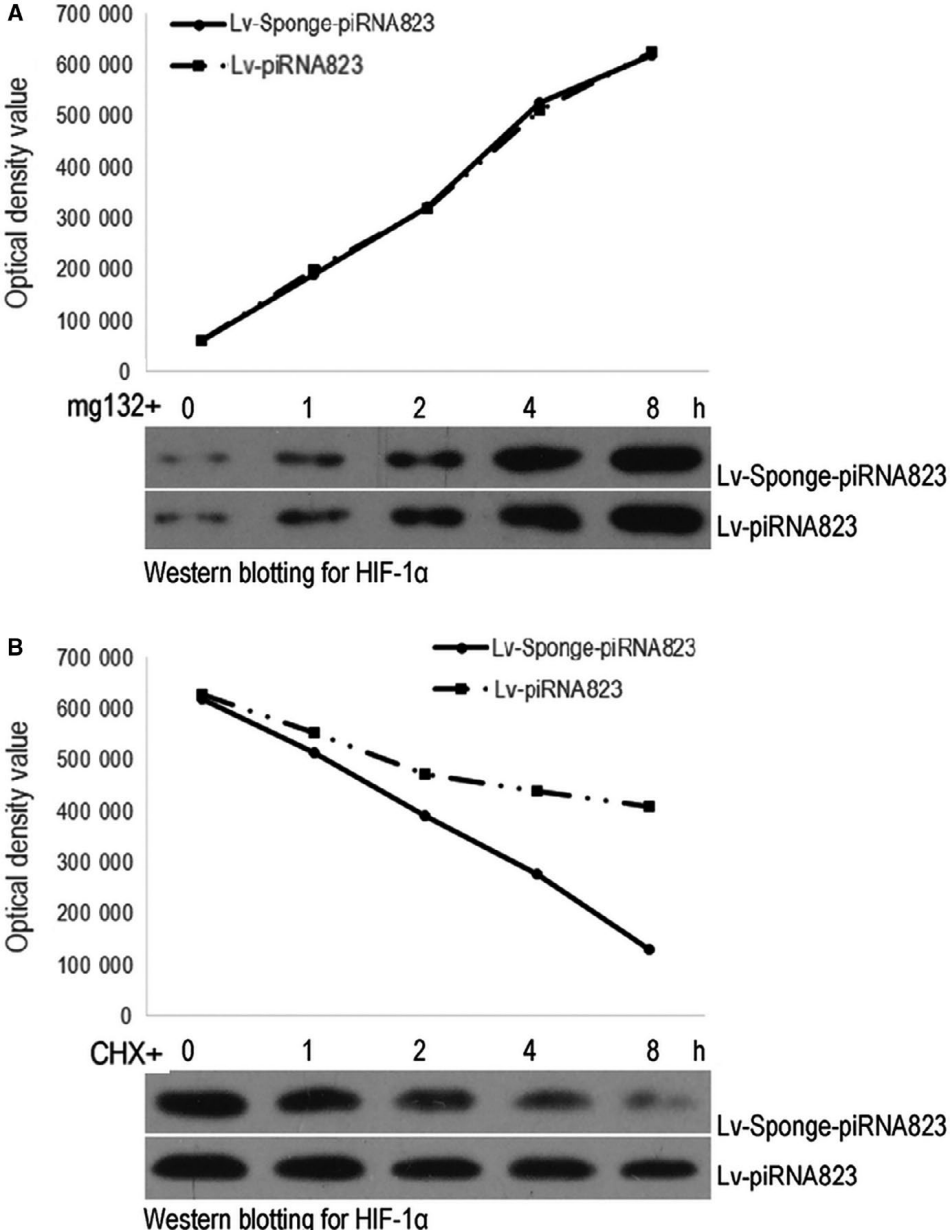
Effect of piRNA‐823 on the ubiquitylation of HIF‐1α in HCT‐116 cells. Protein half‐life detection. A, HIF‐1α change over time after protein degradation was inhibited; B, HIF‐1α change over time after protein synthesis was inhibited

The level of HIF‐1α was increased over time when mg132 suppressed protein degradation in HTC‐116 cells, and there was no difference between the groups infected with Lv‐piRNA‐823 or Lv‐Sponge‐piRNA‐823, indicating piRNA‐823 had no effect on protein synthesis (Figure 6A). When CHX was used to block protein synthesis, HIF‐1α decreased over time in two groups, However, the rate of change with time in Lv‐Sponge‐piRNA‐823‐infected HTC‐116 cells was significantly higher than that of Lv‐piRNA‐823‐infected HCT‐116 cells (Figure 6B).

## 4 DISCUSSION

Colorectal cancer is one of the common malignant tumours, with 930 000 new cases of colorectal cancer every year worldwide, ranking the third among malignant tumours worldwide, and the second among developed countries such as Europe and America.^6^, ^7^, ^8^ Like most malignant tumours, the root cause of colorectal cancer is excessive cell proliferation and severe inhibition of apoptosis, and breaking the dynamic balance between apoptosis and proliferation will lead to the occurrence of cancer and malignant progression of cancer. The process involves abnormal expression of multiple genes and disorder of key signalling pathways, the factors affecting it are intricate and complex, and the relationship between these factors and the molecular mechanism is far from clear. Therefore, it is necessary and possible to discover new drug targets based on the in‐depth study of the key molecular mechanism of the occurrence and development of colorectal cancer. Non‐coding small RNA regulates the expression of target genes at transcriptional and translation levels. Studies have shown that non‐coding small RNAs, including siRNA (small interfering RNA) and microRNA (microRNA), are associated with the occurrence and development of cancer. Some of these small RNAs have extremely important potential value and can become therapeutic targets for cancer diagnosis and treatment.

PiRNA, a newly discovered small molecule RNA, was first found in mammalian germ cells in 2006. PiRNA has a length of 26 ~ 31nt single‐stranded small RNA, most of which are concentrated in 29 ~ 30nt. Like microRNAs, the 5'terminal of piRNA has a strong uracil's tendency (about 86%).^1^ PiRNA corresponds to the genome in a highly specific way and can regulate gene silencing pathways by binding to Piwi subfamily proteins to form piRNA complexes (piRCs). PiRNA was originally thought to play an important role in regulating reproductive system development,^3^ but recent studies have confirmed that piRNA is abnormally expressed in breast cancer, pancreatic cancer, gastric cancer, liver cancer, endometrial cancer and other tumours.^9^, ^10^, ^11^, ^12^, ^13^ In addition, molecular mechanism studies have revealed that piRNA such as piR‐651, piR‐021285, piR‐823 and piR‐Hep1 play important roles in the development of tumours, including the functions of inhibiting or promoting cancer.^14^, ^15^, ^16^, ^17^ The main function of piRNA in cells is to target and silence DNA or RNA containing transposons to maintain genomic stability and ensure the normal function of cells. It is well known that transposons can destroy the structure of coding genes, alter the transcriptional regulatory networks, and cause chromosome breakage or large‐scale gene recombination. Transposons can be divided into two categories: retrotransposons and DNA transposons elements. The retrotransposon can use a mechanism similar to 'copy and paste' to reverse the transcript RNA into a cDNA, which can then be embedded into the genome. DNA transposon elements use the 'cut and past' mechanism to cut one piece of DNA between genomes and insert it into another piece of DNA. Considering that the transposons sequence accounts for nearly 40% of the genome, once such 'jumping genes' are confused, it will lead to genomic devastation. In fact, abnormal transposon regulation has been found to lead to the occurrence of various tumours, including colorectal cancer.^18^ However, in the process of evolution, what mechanism does the cell use to defend the stability of the genome against the interference of 'jumping genes?'. This problem, which has puzzled us for nearly 20 years, was finally solved by the discovery of piRNA.

Although piRNA has not been fully recognized in the occurrence and development of human cancer, the abnormal expression of piRNA in some cancer cells indicates that piRNA may be involved in the occurrence of cancer and may be a potential biomarker for cancer diagnosis and treatment. The research of piRNA in cancer can not only enrich the research content of small RNA but also provide important theoretical basis and application prospects for disease diagnosis, cancer treatment and reproductive regulation in animals and humans in the future. It has far‐reaching significance for the treatment of some diseases, which are difficult to diagnose and treat at present. With the further study of the molecular mechanism of piRNA action, it will provide a new theoretical basis and technical ideas for the development of new RNA diagnosis and treatment technology in cancer treatment in the future. The fundamental cause of colorectal cancer is excessive cell proliferation and severe inhibition of apoptosis, and breaking the dynamic balance between apoptosis and proliferation will lead to the occurrence of cancer and malignant progression of cancer. The process involves abnormal expression of multiple genes and imbalance of key signalling pathways. The factors affecting the occurrence of colorectal cancer are complex and interrelated, and its molecular mechanism is far from clear. Therefore, it is necessary and possible to find new drug targets on the basis of in‐depth study of the key molecular mechanisms of colorectal cancer development. Abnormal expression of piRNA in tumours has been reported in many literatures, which may cause abnormal transposon activity and promote tumour development. In the current study, we found that piRNA‐823 plays an important role in the formation of colorectal cancer, and specific piRNA is involved in the occurrence and development of cancer. Using clinical samples from a large cohorts of CRC patients, we found that overexpression of piRNA‐823 correlated with poor overall survival and predicted a poor response to adjuvant chemotherapy of patients with CRC. These results indicate that piRNA‐823 status in tumours may be a useful tool for estimating CRC patient prognosis and for selecting patients who are likely to benefit from adjuvant chemotherapy to prevent relapse.

This project also aims to elucidate the pathogenic mechanism of piRNA‐823 in the occurrence and development of colorectal cancer through molecular biology experiments and cell function experiments; demonstrate the feasibility of piRNA‐823 as an early diagnosis and clinical prognosis by large‐scale examination of tissue and serum specimens; finally, target silencing of candidate piRNA‐823 was provided therapeutic targets for potential clinical treatment of colorectal patients with cancer. Background investigation showed that as a member of piRNAs, the piRNA‐823 has different expression and function in different types of tumours. The results showed that the expression level of piRNA‐823 in gastric cancer tissues of mice was significantly lower than that in non‐gastric cancer tissues. Increasing the content of piRNA‐823 in gastric cancer cells could significantly inhibit the progress of subcutaneous tumour‐bearing. In addition, the study also found that the content of piRNA‐823 in peripheral blood of patients with gastric cancer was significantly lower than that of healthy people, indicating that piRNA‐823 has the function of tumour suppressor gene in the progress of gastric cancer, and the detection of piRNA‐823 in peripheral blood may be an effective biomarker for gastric cancer.^16^ Yan et al found that piRNA‐823 expression level was increased in multiple myeloma, and silencing piRNA‐823 could induce apoptosis of multiple myeloma cell lines and inhibit their proliferation, suggesting piRNA‐823 has the function of promoting cell proliferation.^13^ Rizzo et al found that the expression of piRNA‐823 was significantly increased in liver cirrhosis, precancerous lesions and hepatocellular carcinoma, suggesting that piRNA‐823 plays a key role in the progression of hepatocellular carcinoma.^19^ However, up to now, the function of piRNA‐823 in colorectal cancer still remains unclear. The aim of this study was to investigate the effects of piRNA‐823 on the proliferation, apoptosis and invasion of colon cancer cell lines HCT‐116 and Lovo, so as to provide theoretical basis for the development of piRNAs‐targeted therapy for colorectal cancer.

G6PD is a key enzyme in pentose phosphate pathway (PPP), and its main function is to provide NADPH in vivo. It is also found that G6PD is highly expressed in many cancer cells and is closely related to some biological characteristics.^20^, ^21^ HIF‐1 plays a key role in the regulation of gene expression induced by hypoxia. HIF‐1α is the only oxygen regulatory subunit, which determines the activity of HIF‐1. HIF‐1a is not only highly expressed in local and metastatic tumours, but also directly related to the differentiation, evolution, invasion, metastasis and prognosis of tumours.^22^, ^23^ Mechanisms studies have shown that HIF‐α can inhibit apoptosis and promote proliferation by down‐regulating ROS in cancer cells.^24^ In the initial stage of the study, we analysed the changes of differentially expressed genes before and after inhibiting the piRNA‐823 by gene expression profiling analysis, and the bioinformatics analysis revealed that the functional genes associated with glucose metabolism pathway significantly increased and decreased in piRNA‐823 overexpressed and inhibited colorectal cancer cells, suggesting that piRNA‐823 may affect the proliferation, invasion and apoptosis of colorectal cancer cells through the regulation of G6PD expression. Especially, it has been reported that G6PD is mainly involved in pentose phosphate pathway, and the lack of G6PD is inversely related to the survival of patients with cancer. Therefore, it is speculated that piRNA‐823 may promote the occurrence and development of colorectal cancer by enhancing the expression of downstream key molecule G6PD.

In the current study, we systematically demonstrated that piRNA‐823 regulates the proliferation, invasion and apoptosis of colon cancer cells by piRNA‐823/G6PD/HIF‐α pathway. On the one hand, piRNA‐823 directly up‐regulates glucose uptake and consumption by up‐regulating G6PD, which may be the direct reason why piRNA‐823 promotes the proliferation of HTC‐116 and Lovo cells. At the same time, G6PD prolongs the half‐life of HIF‐α by inhibiting its ubiquitination and causes HIF‐α to be highly expressed in tumour cells and then decreased the intracellular ROS content immediately, which may be the underlying reason why piRNA‐823 high expression leads to the escape of colorectal cancer cells from death. This study is the first exposition of the piR823/G6PD/HIF‐α regulatory pathway in colorectal cancer and will provide strong support for the development of piRNA‐823‐based gene therapy for colorectal cancer. At the same time, this study also provides a theoretical basis for piRNA‐823 as a promising prognostic biomarker and a potential therapeutic target in colorectal cancer.

## CONFLICT OF INTEREST

The authors declared that they have no financial competing interest.

## AUTHOR CONTRIBUTIONS

**Junlan Feng:** Conceptualization (equal); data curation (lead); formal analysis (lead); methodology (lead); writing‐original draft (lead). **Muqing Yang:** Conceptualization (supporting); methodology (equal); writing‐review and editing (supporting). **Qing Wei:** Conceptualization (equal); methodology (equal); resources (equal). **Feifei Song:** Data curation (equal); formal analysis (equal); methodology (equal). **Youhua Zhang:** Methodology (supporting); resources (equal); writing‐review and editing (supporting). **Xiaodong Wang:** Data curation (supporting); formal analysis (supporting); methodology (supporting); validation (equal). **Bin Liu:** Methodology (equal); resources (supporting); validation (supporting); writing‐original draft (equal). **Jiyu Li:** Conceptualization (lead); funding acquisition (lead); project administration (lead); supervision (lead); writing‐review and editing (lead).

## Supporting information

Table S1

Table S2

## Data Availability

This is an open‐access article under the terms of the Creative Commons Attribution License, which permits use, distribution and reproduction in any medium, provided the original work is properly cited.

## References

[1] Aravin A , Gaidatzis D , Pfeffer S , et al. A novel class of small RNAs bind to MILI protein in mouse testes. Nature. 2006;442:203‐207. 16751777 10.1038/nature04916

[2] Suzuki R , Honda S , Kirino Y . PIWI expression and function in cancer. Frontiers Genetics. 2012;3:204. 10.3389/fgene.2012.00204 PMC3472457 23087701

[3] Hirakata S , Siomi MC . piRNA biogenesis in the germline: From transcription of piRNA genomic sources to piRNA maturation. Biochem Biophys Acta. 2016;1859:82‐92. 26348412 10.1016/j.bbagrm.2015.09.002

[4] Li YY , Wu HS , Tang L , et al. The potential insulin sensitizing and glucose lowering effects of a novel indole derivative in vitro and in vivo. Pharmacol Res. 2007;56:335‐343. 17889553 10.1016/j.phrs.2007.08.002

[5] Shanker G , Aschner M . Methylmercury‐induced reactive oxygen species formation in neonatal cerebral astrocytic cultures is attenuated by antioxidants. Brain Res Mol Brain Res. 2003;110:85‐91. 12573536 10.1016/s0169-328x(02)00642-3

[6] Chen W , Zheng R , Baade PD , et al. Cancer statistics in China, 2015. CA: Cancer J Clinicians. 2016;66:115‐132. 10.3322/caac.21338 26808342

[7] Siegel RL , Miller KD , Jemal A . Cancer statistics, 2018. CA: Cancer J Clinicians. 2018;68:7‐30. 10.3322/caac.21442 29313949

[8] Favoriti P , Carbone G , Greco M , Pirozzi F , Pirozzi RE , Corcione F . Worldwide burden of colorectal cancer: a review. Updates Surgery. 2016;68:7‐11. 10.1007/s13304-016-0359-y 27067591

[9] Muller S , Raulefs S , Bruns P , et al. Next‐generation sequencing reveals novel differentially regulated mRNAs, lncRNAs, miRNAs, sdRNAs and a piRNA in pancreatic cancer. Mol Cancer. 2015;14:94. 25910082 10.1186/s12943-015-0358-5 PMC4417536

[10] Martinez VD , Enfield KSS , Rowbotham DA , Lam WL . An atlas of gastric PIWI‐interacting RNA transcriptomes and their utility for identifying signatures of gastric cancer recurrence. Gastric Cancer. 2016;19:660‐665. 25779424 10.1007/s10120-015-0487-y PMC4573768

[11] Ravo M , Cordella A , Rinaldi A , et al. Small non‐coding RNA deregulation in endometrial carcinogenesis. Oncotarget. 2015;6:4677‐4691. 25686835 10.18632/oncotarget.2911 PMC4467107

[12] Huang G , Hu H , Xue X , et al. Altered expression of piRNAs and their relation with clinicopathologic features of breast cancer. Clin Transl Oncol. 2013;15:563‐568. 23229900 10.1007/s12094-012-0966-0

[13] Cui L , Lou Y , Zhang X , et al. Detection of circulating tumor cells in peripheral blood from patients with gastric cancer using piRNAs as markers. Clin Biochem. 2011;44:1050‐1057. 21704610 10.1016/j.clinbiochem.2011.06.004

[14] Fu A , Jacobs DI , Hoffman AE , Zheng T , Zhu Y . PIWI‐interacting RNA 021285 is involved in breast tumorigenesis possibly by remodeling the cancer epigenome. Carcinogenesis. 2015;36:1094‐1102. 26210741 10.1093/carcin/bgv105 PMC5006152

[15] Wu D , Fu H , Zhou H , Su J , Zhang F , Shen J . Effects of novel ncRNA molecules, p15‐piRNAs, on the methylation of DNA and histone H3 of the CDKN2B promoter region in U937 cells. J Cell Biochem. 2015;116:2744‐2754. 26205624 10.1002/jcb.25199

[16] Yan H , Wu QL , Sun CY , et al. piRNA‐823 contributes to tumorigenesis by regulating de novo DNA methylation and angiogenesis in multiple myeloma. Leukemia. 2015;29:196‐206. 24732595 10.1038/leu.2014.135

[17] Cheng J , Deng H , Xiao B , et al. piR‐823, a novel non‐coding small RNA, demonstrates in vitro and in vivo tumor suppressive activity in human gastric cancer cells. Cancer Lett. 2012;315:12‐17. 22047710 10.1016/j.canlet.2011.10.004

[18] Guo M , Wu Y . Fighting an old war with a new weapon–silencing transposons by Piwi‐interacting RNA. IUBMB Life. 2013;65:739‐747. 23893818 10.1002/iub.1192

[19] Rizzo F , Hashim A , Marchese G , et al. Timed regulation of P‐element‐induced wimpy testis‐interacting RNA expression during rat liver regeneration. Hepatology. 2014;60:798‐806. 24930433 10.1002/hep.27267

[20] Ohl F , Jung M , Radonic A , Sachs M , Loening SA , Jung K . Identification and validation of suitable endogenous reference genes for gene expression studies of human bladder cancer. J Urol. 2006;175:1915‐1920. 16600798 10.1016/S0022-5347(05)00919-5

[21] Zhang X , Zhang X , Li Y , et al. PAK4 regulates G6PD activity by p53 degradation involving colon cancer cell growth. Cell Death Dis. 2017;8:e2820. 28542136 10.1038/cddis.2017.85 PMC5520749

[22] Swinson DE , Jones JL , Cox G , Richardson D , Harris AL , O'Byrne KJ . Hypoxia‐inducible factor‐1 alpha in non small cell lung cancer: relation to growth factor, protease and apoptosis pathways. Int J Cancer. 2004;111:43‐50. 15185341 10.1002/ijc.20052

[23] Semenza GL . Development of novel therapeutic strategies that target HIF‐1. Expert Opin Ther Targets. 2006;10:267‐280. 16548775 10.1517/14728222.10.2.267

[24] Chun SY , Johnson C , Washburn JG , Cruz‐Correa MR , Dang DT , Dang LH . Oncogenic KRAS modulates mitochondrial metabolism in human colon cancer cells by inducing HIF‐1alpha and HIF‐2alpha target genes. Mol Cancer. 2010;9:293. 21073737 10.1186/1476-4598-9-293 PMC2999617

