# Supplementary material for: Novel evidence for oncogenic piRNA‐823 as a promising prognostic biomarker and a potential therapeutic target in colorectal cancer
Source: J Cell Mol Med. 2020 Jun 28;24(16):9028–40. doi: 10.1111/jcmm.15537 (PMC7417729; doi:10.1111/jcmm.15537)
Supplement: Supplementary file 1 — Table S1 [file JCMM-24-9028-s001.docx]

**Table S1. A detailed clinical information for 176 colorectal cancer patients.**

| Clinical features | Number (%)^a^ |
| --- | --- |
| Sex – no. (%) |  |
| Female | 80 (45.45) |
| Male | 96 (54.55) |
| Age – year |  |
| Median | 67 |
| Range | 31-87 |
| Location – no. (%)^b^ |  |
| Ascending colon | 54 (30.68) |
| Transverse colon | 8 (4.55) |
| Descending colon | 18 (10.23) |
| Sigmoid colon | 46 (26.14) |
| Rectum | 49 (27.84) |
| Missing data | 5 (2.84) |
| TNM stage – no. (%)^c^ |  |
| I | 11 (6.25) |
| II | 94 (53.41) |
| IIA | 46 (26.14) |
| IIB | 48 (27.27) |
| III | 55 (31.25) |
| IIIA | 2 (1.14) |
| IIIB | 35 (19.89) |
| IIIC | 18 (10.23) |
| IV | 9 (5.11) |
| Missing | 7 (3.98) |
| Pathological type – no. (%) |  |
| Tubular adenocarcinoma | 142 (80.68) |
| Mucinous adenocarcinoma | 20 (11.36) |
| Mixed adenocarcinoma | 14 (7.95) |
| Tumor size – no. (%) |  |
| < 5cm | 87 (49.43) |
| ≥ 5cm | 68 (38.64) |
| Missing data | 21 (11.93) |
| Lymph node metastasis – no. (%) |  |
| Yes | 62 (35.23) |
| No | 114 (64.77) |
| Missing data | 0 (0) |
| Differentiation –no. (%) |  |
| Well | 9 (5.11) |
| Moderate | 127 (72.16) |
| Poor | 40 (22.73) |
| Adjuvant chemotherapy – no. (%) |  |
| Yes | 60 (34.09) |
| No | 108 (61.36) |
| Missing data | 8 (4.55) |
| Survival – no. (%) |  |
| Median | 67.97 |
| Range | 0.17 – 110.90 |

^a^ Percentages may not sum to 100 due to rounding.

^b^ Tumor location was available for 175 patients, 4 patients were suffered with both of rectal cancer and sigmoid colon cancer.

^c^ TNM denotes tumor-node-metastasis.
